# Optical Coherence Tomography Reveals Sigmoidal Crystalline Lens Changes during Accommodation

**DOI:** 10.3390/vision2030033

**Published:** 2018-08-21

**Authors:** George A. Gibson, Fiona E. Cruickshank, James S. Wolffsohn, Leon N. Davies

**Affiliations:** Ophthalmic Research Group, Life and Health Sciences, Aston University, Birmingham B4 7ET, UK

**Keywords:** accommodation, crystalline lens, imaging, in-vivo, optical coherence tomography

## Abstract

This study aimed to quantify biometric modifications of the anterior segment (AS) during accommodation and to compare them against changes in both accommodative demand and response. Thirty adults, aged 18–25 years were rendered functionally emmetropic with contact lenses. AS optical coherence tomography (AS-OCT) images were captured along the 180° meridian (Visante, Zeiss Meditec, Jena, Germany) under stimulated accommodative demands (0–4 D). Images were analysed and lens thickness (LT) was measured, applying a refractive index correction of 1.00. Accommodative responses were also measured sequentially through a Badal optical system fitted to an autorefractor (Shin Nippon NVision-K 5001, Rexxam, Japan). Data were compared with Dubbelman schematic eye calculations. Significant changes occurred in LT, anterior chamber depth (ACD), lens centroid (i.e., ACD + LT/2), and AS length (ASL = ACD + LT) with accommodation (all *p* < 0.01). There was no significant change in CT with accommodation (*p* = 0.81). Measured CT, ACD, and lens centroid values were similar to Dubbelman modelled parameters, however AS-OCT overestimated LT and ASL. As expected, the accommodative response was less than the demand. Interestingly, up until approximately 1.5 D of response (2.0 D demand), the anterior crystalline lens surface appears to be the primary correlate. Beyond this point, the posterior lens surface moves posteriorly resulting in an over-all sigmoidal trajectory. he posterior crystalline lens surface demonstrates a sigmoidal response with increasing accommodative effort.

## 1. Introduction

Accommodative refractive change is primarily brought about by alterations in the surface curvatures of the crystalline lens. During accommodation, the anterior radius of curvature decreases [1,2,3], accompanied by a smaller simultaneous steepening of the posterior surface [4,5,6]. The lens equatorial diameter decreases [7,8,9,10,11,12,13,14,15], with the associated curvature changes resulting in a reduction of the anterior chamber depth [1,16,17,18,19,20,21,22] and an increase in lens axial thickness [1,5,16,17,18,19,20,21,23,24,25,26,27]. Crystalline lens surface curvature changes are the primary cause of dioptric change with accommodation [3,19]. Alterations in axial surface distances (i.e., the reduction in anterior chamber depth (ACD) and increase in crystalline lens thickness (LT)) appear to reduce accommodative response with increasing levels of accommodative demand [5,28].

A number of different methods have been employed to quantify intraocular surface distances. A-scan ultrasonography has been used to measure static changes in ocular biometry in humans and primates, the latter using Edinger-Westphal (EW) and pharmacological stimulation [1,29,30,31,32,33]. Dynamic changes in anterior segment (AS) biometry have also been studied in humans and primates using continuous high-resolution ultrasonography [9,20,29,33,34,35,36,37,38,39,40,41,42,43]. Other imaging techniques are also capable of quantifying ocular biometric changes associated with accommodation. These include Scheimpflug photography [1,2,19,44,45,46,47], magnetic resonance imaging (MRI) [8,12,24], partial coherence interferometry (PCI) [17,21,48], and AS optical coherence tomography (AS-OCT) [26,49,50,51]. Hitherto, AS-OCT has been used to measure anterior chamber depth, width and angle [49,52,53,54,55,56,57], to evaluate phakic intraocular lens implants [58,59,60] and to determine corneal integrity [61,62,63]. The accuracy of this ocular biometric data is also important in determining the risk of angle closure glaucoma, and for pre- and post-operative assessment in cataract and kerato-refractive surgery [53].

AS-OCT also allows measurement of ocular biometric changes associated with the accommodative response. Richdale et al. [26] showed that lens thickness changes during accommodation as measured with the Visante AS-OCT (Carl Zeiss Meditec, Dublin, CA, USA) are comparable to previous findings using Scheimpflug photography [18], ultrasound [4], MRI [12] and PCI [21,64]. However, a limitation of this work is that biometric alterations have been plotted against accommodative stimulus and not response [26], which may have led to erroneous conclusions. Hence, this study aims to quantify AS changes during accommodation in a cohort of young participants. Importantly, and in contrast to previous work, biometric modifications are compared against changes in both accommodative demand and response. In addition, comparisons are made with data from a well-validated schematic model eye [65] based on empirical work acquired with Scheimpflug photography [45,46,47]. A mean age of 19.4 years has been used for Dubbelman values.

## 2. Materials and Methods 

Thirty participants from Aston University were recruited, aged 18 to 25 years (mean ± SD, 19.4 ± 2.0 years) 40% of whom were male. Mean spherical equivalent (MSE; sphere + [cylinder/2]) was −1.85 D ± 2.68 D (range: +1.94 D to −6.87 D). Participants with astigmatism ≥1.0 D were excluded from the study. All participants achieved 0.0 logMAR visual acuity or better in the eye tested, with a subjective amplitude of accommodation ≥8.0 D (RAF gauge, Clement Clarke/Haag-Streit, Harlow, UK). No participant had any form of visual or pathological anomaly. Participants were furnished with a full explanation of the procedures involved in the investigation, and gave informed consent to their participation, in line with the tenets of the Declaration of Helsinki. Ethical approval was granted by the Human Ethics Committee of Aston University.

Uncorrected distance autorefraction was performed on each participant using the previously validated Shin-Nippon *NVision-K 5001* (Shin-Nippon Commerce Inc., Tokyo, Japan) open view autorefractor [66]. Slit lamp examination was performed on all participants to assess anterior eye health and suitability for contact lens wear, before participants were rendered functionally emmetropic with conventional daily disposable spherical soft contact lenses (etafilcon A, 58% water content, Johnson & Johnson Vision, Jacksonville, FL, USA) to ensure that the accommodative demand was virtually identical for each subject. Sufficient time (approximately 15 min) was given for adaptation to the soft contact lenses before assessment. All participants were existing soft contact lens wearers, and no participant experienced difficulties. All participants were experienced with visual experiments and, prior to the full study, were trained to maintain steady fixation.

Initially, the residual refractive error of each participant was measured whist viewing a Maltese cross (contrast: 78%; luminance: 32 cdm^−2^) through a +5.0 D Badal optical system with the target placed at 0.0 D. Participants were excluded if their MSE residual refractive error exceeded ±0.25 D, or if the residual cylindrical component was >0.50 DC. With the left eye occluded, the right eye of each participant viewed the stationary Maltese cross target through the Badal optical system, fitted to the autorefractor. Five accommodative stimulus levels were presented in a random order within the Badal system (0.0 D to 4.0 D in 1.0 D steps). Participants were encouraged to “carefully focus” [67] on the target at all times, as attentional factors can influence accommodation measurement [68]. Five measures of the participant’s accommodation response were recorded at each stimulus level and averaged to provide a stimulus-response profile. 

To measure the associated biometric changes in crystalline lens thickness and position, participants were asked to place their head on the automated chin and headrest of the AS-OCT (Visante OCT, model 1000, Carl Zeiss Meditec, Dublin, CA, USA) so that the captured image of the eye was central in the integral computer monitor window. Again, participants were encouraged to concentrate on the internal pinwheel target at all times. Two separate images were captured along the 180° meridian in the AS single-image capture mode. According to the instrument’s user manual, this setting provides an axial resolution of approximately 18 µm and a transverse resolution of 60 µm with an axial scan accuracy of ±15 µm (1 SD) over 6 mm. The device employs low-coherence interferometry, via a superluminescent light-emitting diode (central wavelength 1310 nm, optical power <6.5 mW at the cornea). The scanning spot moves rapidly across the eye, acquiring 2000 A-scans per second, with a scan time of 0.125 s per line in anterior segment mode, to generate a two-dimensional image which covers an area of 16 mm in width and 6 mm in depth. 

**Figure 1:**
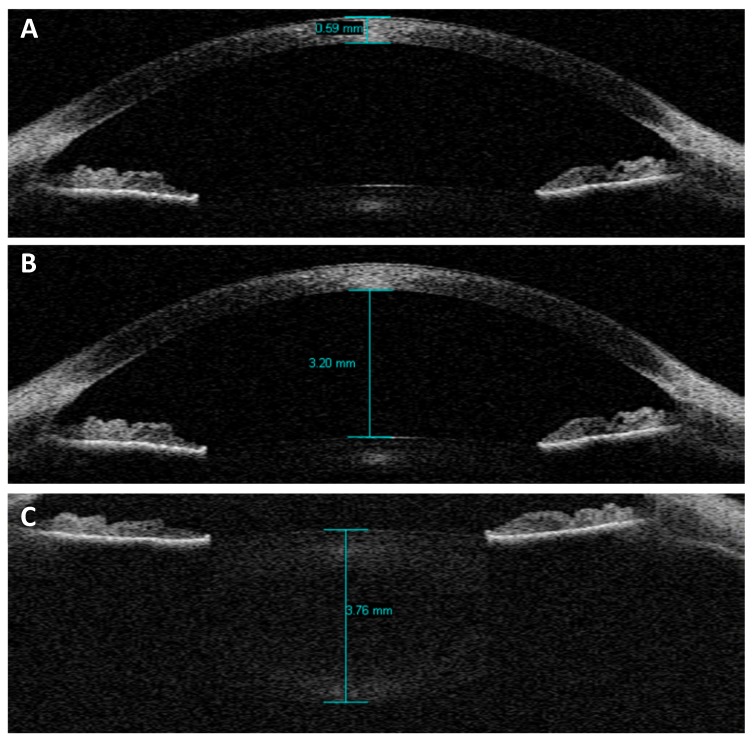
The Visante AS-OCT screen displaying measuring calipers in situ for measurement of (**A**) corneal thickness (CT), (**B**) anterior chamber depth (ACD) and (**C**) lens thickness (LT). Images are not shown for anterior segment length (ASL) or lens centroid (LC) as these values are derived by calculation from CT, ACD, and LT measures. Note that though CT and ACD are drawn on separate images here for demonstration purposes, during real data analysis calipers were drawn simultaneously on the same image using the software’s function to hide from view calipers which obscure the region required for subsequent measures.

The first image included corneal thickness and anterior chamber depth (Figure 1A,B respectively). The second image involved changing the focal plane of the AS-OCT, enabling measurement of crystalline lens thickness (Figure 1C). As with the autorefractor measures, participants were exposed to five stimulus levels, presented in a random order, using the AS-OCT’s internal pinwheel target and the internal adjustable Badal lens system. This process was repeated three times to generate mean AS changes. 

Image analysis was performed using the Visante‘s built-in software (version 1.0.12.1896, Carl Zeiss Meditec, Dublin, CA, USA). The software has an inbuilt refractive indices adjustment feature which uses edge detection algorithms to locate corneal surfaces and automatically assign appropriate indices to each region of the image and, therefore, automatically scale individual structural dimensions [69]. A refractive index of 1.00 (air) is applied to the region anterior to the cornea, 1.338 (cornea) for the region within the corneal boundaries, and 1.343 (aqueous humour) for structures posterior to the cornea. However, when imaging the crystalline lens, the cornea is not simultaneously visible; therefore, appropriate dimensional adjustments cannot be made. Consequently, a refractive index of 1.00 was applied to the entirety of all images using the Edit Surfaces option, before measurements were taken, as in previous studies that have examined internal ocular structures without a simultaneous corneal image [69].

All images were analysed by the same examiner (G.A.G.). Following refractive index adjustment, intraocular distances were measured using the built-in Visante measuring calipers. Measurement involved the manual placement of the endpoint of each caliper arm on the boundary edge of the relevant anterior segment structural landmark. Corneal thickness (CT) was defined as the anteroposterior distance between the cornea’s front-most and back-most surfaces (Figure 1A), anterior chamber depth (ACD) as the distance between the posterior corneal surface and the anterior crystalline lens surface (Figure 1B) and lens thickness (LT) as the distance between the anterior crystalline lens surface and the posterior crystalline lens surface (Figure 1C). Where necessary, adjustments to brightness and contrast settings were made using the software’s built-in capabilities, to facilitate localisation of the relevant structural boundaries. Furthermore, the software allows the operator to manually adjust the image magnification to enhance the accuracy of caliper placement. Lens centroid (LC = ACD + LT/2) and anterior segment length (ASL = ACD + LT; the position of the posterior crystalline lens surface) were then calculated from ACD and LT measures. All distances were measured three separate times. Means and standard deviations were calculated using a Microsoft Excel spreadsheet.

The Dubbelman eye model is a schematic eye derived by Norrby [65], and is based on the work of Dubbelman and colleagues, who collected substantial biometric data using Scheimpflug photography [45,46,47]. The model incorporates aspheric surfaces and can be used to compute predicted intraocular biometric dimensions and spacings. Equations were based on the same group of 102 eyes in which the mean refractive error was −1.10 D, including 42 myopes, 47 near emmetropes and 13 hyperopes. Participants were aged 16–65 years (mean 39.2 years) [45,46,47]. The Dubbelman Eye Model has been chosen to validate the crystalline lens measures in the current study, as it can be altered for both age (A) and accommodation demand (D) (as all of the studies used to derive the Model were based on accommodative demand rather than accommodative response). Refractive parameters and the formulae used to derive biometrics from the Dubbelman eye model are shown in Table 1. For the current study, values for CT, ACD, and LT were calculated from these formulae with an inputted age (A) of 19.4 years to match the average age of the participant cohort. Calculations were performed at accommodation levels of 0.00 to 4.00 dioptres in steps of 1.0 D to provide a modelled value to match the data for each stimulus level collected from the participants. LC and ASL values were calculated from the ACD and LT values modelled for the same stimulus level, using the same equations as used for participant data. 

### Statistical Analysis

A repeated measures analysis of variance (ANOVA) [70] was conducted with SPSS 12.0.1 for Microsoft Windows (SPSS Inc, Chicago, IL, USA), where accommodation demand was taken as the within-subject variable. A sample size calculation using G*Power 3 [71] was based on a repeated measures ANOVA, with an effect size (f) of 0.25, an error probability (α) of 0.05, and required power (1 – β) of 0.90 for five repeated measurements, indicated 26 participants were required.

## 3. Results

The mean magnitude of change during accommodation as measured by the AS-OCT was similar to the Dubbleman eye model [65]. Moreover, the measures for CT, ACD, LT, LC, and ASL were also similar (Table 2). The largest discrepancy between approaches appears to be LT and ASL, where the AS-OCT appears to overestimate these components in comparison to the Scheimpflug data described by Dubbelman [19,45,46,47].

Some studies [5,21,49], which have assessed AS biometric changes, consider the ACD to be the distance between the anterior vertex of the cornea and the anterior vertex of the crystalline lens, thus including the thickness of the cornea. The absolute values displayed in Table 2, therefore, require the addition of the corneal thickness to allow comparisons to be made across studies. For example, the mean ACD of the Dubbelman model at rest (0.0 D stimulus) is 3.67 mm. A similar calculation must be made when considering LC and ASL; these corrected data are also shown in Table 2. 

**Figure 2:**
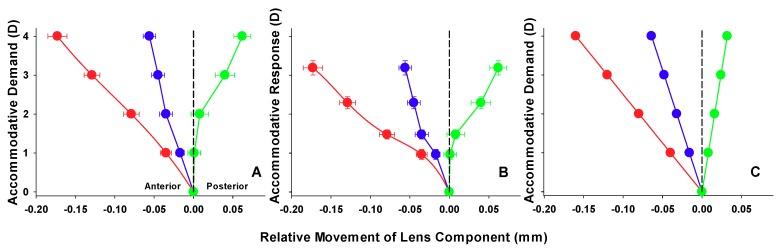
Relative movements of the various lens parameters as a function of accommodative demand or response. Red markers represent the anterior lens surface, blue markers the lens centroid and green markers the posterior lens surface. (**A**) The relative movement of the lens components for the AS-OCT data with accommodative demand (error bars represent ±SEM). (**B**) The relative movement of the lens components for the AS-OCT data with accommodative response (±SEM). (**C**) The relative movement of the lens components for the Dubbelman model eye [65] with accommodative demand.

As the studies by Dubbelman and colleagues rely on measures attributed to accommodative demand [19,45,46,47], a degree of error may be inherent in the model, as the axial distances and relative changes given may be related to a different accommodative response. Figure 2 illustrates, as expected, the accommodative response is less than that of demand, due to the lag of accommodation. What is also of interest is that the lens changes appear to be sigmoidal. Up until approximately 1.5 D of response (approximately 2.0 D demand), the anterior crystalline lens surface appears to be the primary correlate. After this point, the posterior surface begins to alter position. This is contrary to the model data (Figure 2C), which predict a linear pattern of lens changes.

When comparing the relative changes in axial distances, the two methods (Scheimpflug and AS-OCT) appear to be more closely associated (Table 3). These axial measures are very similar, with the AS-OCT data falling very close to the model distances. Corneal thickness did not change with accommodation (F _(4,29)_ = 0.40; *p* = 0.81). All other measures, however, reached statistical significance (ACD: F _(4,29)_ = 116.96; *p* < 0.01, LT: F _(4,29)_ = 128.97; *p* < 0.01, LC: F _(4,29)_ = 17.30; *p* < 0.01, ASL: F _(4,29)_ = 13.67; *p* < 0.01). When the AS-OCT distances were corrected to include CT, the accommodative changes remained significant (ACD: F _(4,29)_ = 117.56; *p* < 0.01, LC: F _(4,29)_ = 17.45; *p* < 0.01, ASL: F _(4,29)_ = 14.72; *p* < 0.01). 

## 4. Discussion

Understanding the changes in anterior segment geometry with accommodation is critical to understanding fully the lenticular mechanism of accommodation. AS-OCT is an important technological advance, which has provided a non-invasive means of accurately visualising the performance and morphology of the accommodative apparatus and its associated biometric modifications in vivo. As such, some of the uncertainties previously attributed to the difficulties of obtaining direct measures of the accommodative apparatus’ elements as they work in concert are now being illuminated. However, a limitation of some previous work is a tendency to consider biometric modifications in terms of accommodative demand in isolation, rather than a measured response. This study provides estimates of the ocular biometry associated with accommodation in the adult human eye, with reference to both accommodative demand and response and, furthermore, compares them with those predicted from accommodating model eye calculations [65].

The key accommodative changes in anterior segment biometry in the present investigation are a reduction in ACD, an increase in LT, a reduction in LC, and an increase in ASL, while corneal thickness was unchanged (Table 2), all consistent with the Helmholtzian model of accommodation. In the accommodated state (4 D stimulus) LT increased by 0.24 mm compared to in the unaccommodated state, consistent with previous reports [1,5,16,17,18,19,20,21,23,24,25,26,27], while there was a concurrent 0.06 mm reduction in lens centroid and 0.061 mm increase in ASL. Furthermore, this study reports agreement between axial separations measured on AS-OCT images, and those predicted from accommodating model eye calculations [65]. However, the lens thickness and, subsequently, ASL values appear to be overestimated by AS-OCT compared to the calculated figures (Table 3); a finding which is corroborated by the work of Dunne and colleagues [72].

Measured ACD was significantly shallower (0.17 mm) in the unaccommodated state compared to under 4.00 D of accommodative stimulus, in accordance with previous in vivo reports [1,16,17,18,19,20,21,22]. Baikoff et al. [49] found a mean reduction in ACD length of 30 μmD^−1^ using AS-OCT, comparable to the present study’s measured and modelled values of 34 μmD^−1^ and 40 μmD^−1^, respectively. Ostrin et al. [20] found this decrease to be greater, in the region of 51 μmD^−1^ using A Scan and a young cohort of between 21 and 30 years, whereas Bolz et al. [64] found a decrease of 47 μmD^−1^ in emmetropes and 57 μmD^−1^ in myopes using PCI.

The current study reports a 58 μmD^−1^ rate of change in LT with accommodation, comparable to the work of Richdale and colleagues [26] who found a 51 μmD^−1^ increase, also using AS-OCT. The results from both studies are greater than those predicted by the Dubbelmann model (45 μmD^−1^). Other studies, which have determined LT based on PCI, have found variable thickness changes, in the order of 36 μmD^−1^ [21], and in a separate study, 63 μmD^−1^ for emmetropes and 72 μmD^−1^ for myopes [64]. An ultrasound study has reported a lower value of 42 μmD^−1^ [4]. It must be stressed that the stimuli used these studies inevitably vary with instrumentation and study group. Alterations in axial surface distances (i.e., the reduction in anterior chamber depth (ACD) and the increase in crystalline lens thickness (LT)) appear to reduce accommodative response with increasing levels of accommodative demand [5,28]. 

Perhaps the most interesting and novel finding of this study is the sigmoidal response of the posterior crystalline lens surface during accommodation; a phenomenon so far unreported in the literature, with previous human and animal studies finding an equal and linear change in the anterior and posterior portions of the lens with accommodation [1,29]. In the current study, the posterior surface appears to be relatively static until approximately 1.5 D of response (approximately 2.0 D demand), after which it begins to make its posterior motion (Figure 2B). Drexler and colleagues [17] plotted the position of the anterior and posterior poles of the crystalline lens with change in fixation from far viewing to targets at various closer distances. The results show a static posterior lens surface from distance observation until viewing a target at 40.0 cm (2.5 D stimulus). From this point, closer targets elicit a posterior movement of the surface of just over 0.5 mm for a target at 10.0 cm (10.0 D stimulus); this phenomenon is, however, not alluded to in the text; it appears to be relatively new in the study of accommodative lenticular changes. What is well established from other work is that the changes in lenticular radius during accommodation are greater for the anterior surface of the lens [1,2,3,5]. The initial absence of movement of the posterior lens surface to low accommodative stimuli, may, perhaps result from a mechanical resistance exerted by the vitreous body. In combination with the forward movement of the anterior lens surface, there is a resulting increase in axial thickness and a small forward movement of the LC [17,23,24]. This may also explain why there is a similar, though much less pronounced sigmoidal response also seen for the anterior lens surface, potentially due to a similar resistance to forward movement by the aqueous humour. The relatively lower magnitude of the initial inertia may be due to the relatively smaller volume of humour against the lens’ anterior face and therefore less mechanical restriction. It is known that the vitreous body undergoes a syneresis with age [73], and therefore, it could be hypothesised that the posterior surface sigmoidal response may undergo attenuation with age as vitreous resistance lessens due to liquefaction. This again may have contributed a lack of sigmoidal response in the Dubbleman data and other such studies which have older or wide age inclusion criteria. It can be seen from our data that the shape of the measured functions are noticeably different between demand and response for anterior lens surface, posterior lens surface and lens centroid (Figure 2). Though the sigmoidal response can still be inferred from demand data, it is markedly less pronounced than shown for response. It may follow that biometric studies which fail to consider accommodative responses may undergo a masking of such accommodative trends.

The findings of the current study notwithstanding, AS-OCT imaging does have some important limitations which are relevant to this study. Firstly, as with other two-dimensional imaging techniques [8], it is difficult to ensure that sequential AS-OCT slices are taken from the same plane. To mitigate this, the patient’s forehead was in contact with the forehead rest at all times, and multiple images were acquired and averaged for each accommodative level. A further potential limitation associated with referencing an appropriate image slice plane concerns the putative cyclotorsion effects observed during accommodation [74] although this remains equivocal [75]. Although the Visante allows variation in the meridian of the image slice, this is only in one degree steps, which is insufficient to compensate for the small amounts of cyclotorsion associated with accommodation where mean cyclotorsion has been reported as 0.62 ± 2.18 degrees excyclorotation for an accommodative stimulus up to 6.0 D [74]. 

Lack of image correction for optical and instrument distortion may also be of importance. The application of a standard refractive index (1.00) across both AS-OCT images was a necessary adjustment to ensure the comparability of within subject biometric measures. However, other studies using OCT imaging have utilized a system wherein separately collected corneal and lenticular images can be merged and corrected after capture [15,76]. The use of such a technique was not possible in the current study owing to the use of commercial, non-custom analysis software. Furthermore, the Visante, like other optical imaging techniques, assumes a single refractive index of the lens [12,26] (i.e., 1.42 [77,78]) and does not take into account the gradient refractive index (GRIN) of the crystalline lens as reported by other authors [12,45,76]. Studies have also specifically examined the correction of the posterior surface with different homogeneous refractive indices, and also using a GRIN [79,80], and have reported that for both thickness and shape measurement the best approach was to use an average refractive index (*n* = 1.408 ± 0.005) as defined by Uhlhorn and colleagues [81]. Though the precise effect of not applying such a correction factor on the accuracy of the present study is unknown, relative changes in axial separation of the ocular components were compared on the assumption that any distortion or manipulation of the image by the AS-OCT is constant throughout the image taken, thereby allowing comparison with other techniques. In this instance, the data points from the OCT and from the model eye are very close, with all model data falling well within the compass of any errors (SD) from the AS-OCT cohort.

## 5. Conclusions 

The study reveals a sigmoidal response of the posterior lenticular surface with increasing accommodative effort; a phenomenon which has hitherto gone unreported in previous studies. Additionally, the use of axial distance measures acquired directly by AS-OCT compares well with a published schematic eye model [65]. Despite this, some caution should be taken when applying such linear models to infer the biometric corollaries of low accommodative stimuli, as they may not entirely reflect the true nature of the behaviour of the ocular structures. The majority of studies of this type relate biometric changes to the accommodative stimulus as opposed to the response. Although it is difficult to inextricably link accommodative demand and response, the latter would suggest a better evaluation of actual accommodative performance.

## Figures and Tables

**Table 1:** The Dubbelman eye model [65]. Component radii, axial thicknesses, and refractive indices are shown. The equations shown refer to the dependence of various components on years of age (A) and dioptres of accommodative demand (D).

Component	Parameter
Cornea	
Anterior radius (mm)	7.87
Thickness (mm)	0.574
Refractive index	1.376
Posterior radius (mm)	6.40
Anterior Chamber	
Depth (mm)	3.87 − 0.010A − D (0.048 − 0.0004A)
Refractive index	1.336
Crystalline Lens	
Anterior radius (mm)	1/[1/(12.7 − 0.058A) + 0.0077D]
Thickness (mm)	2.93 + 0.0236A + D (0.058 − 0.0005A)
Refractive index	1.441 − 0.00039A + 0.0013D
Posterior radius (mm)	1/[1/(5.9 − 0.0013A) + 0.0043D]
Vitreous	
Depth (mm)	Variable (see text)
Refractive index	1.376

**Table 2:** Anterior eye axial distances and their variation with accommodation demand measured with the AS-OCT, compared to the Dubbelman model eye data [65]. The average age of the cohort has been matched to that of the model (19.4 years old; *n* = 30). The ACD and subsequent measures have been altered to include the corneal thickness where required. To note, correction for CT is not required for measures of LT alone.

Accommodation Demand (D)	Dubbelman Model (mm)	Dubbelman Model (mm) Adjusted for CT	AS-OCT (mm ± SD)	AS-OCT (mm ± SD) Adjusted for CT
	Corneal Thickness (CT)			
0	0.574	-	0.551 ± 0.030	-
1	0.574	-	0.552 ± 0.033	-
2	0.574	-	0.553 ± 0.029	-
3	0.574	-	0.554 ± 0.029	-
4	0.574	-	0.552 ± 0.033	-
	Anterior Chamber Depth (ACD)			
0	3.096	3.670	3.102 ± 0.280	3.653 ± 0.277
1	3.056	3.630	3.066 ± 0.287	3.618 ± 0.285
2	3.016	3.590	3.021 ± 0.287	3.574 ± 0.286
3	2.976	3.550	2.970 ± 0.283	3.524 ± 0.278
4	2.936	3.510	2.928 ± 0.282	3.480 ± 0.280
	Lens Thickness (LT)			
0	3.402	-	3.632 ± 0.205	-
1	3.450	-	3.669 ± 0.200	-
2	3.498	-	3.719 ± 0.210	-
3	3.546	-	3.802 ± 0.226	-
4	3.594	-	3.867 ± 0.219	-
	Lens Centroid (ACD + LT/2)			
0	4.797	5.371	4.918 ± 0.235	5.469 ± 0.232
1	4.781	5.355	4.900 ± 0.235	5.452 ± 0.233
2	4.765	5.339	4.881 ± 0.239	5.434 ± 0.237
3	4.749	5.323	4.871 ± 0.232	5.425 ± 0.229
4	4.733	5.307	4.861 ± 0.228	5.413 ± 0.226
	Anterior segment Length (ACD + LT)			
0	6.498	7.072	6.734 ± 0.230	7.285 ± 0.229
1	6.506	7.080	6.735 ± 0.220	7.287 ± 0.218
2	6.514	7.088	6.740 ± 0.231	7.294 ± 0.230
3	6.522	7.096	6.772 ± 0.232	7.326 ± 0.231
4	6.530	7.104	6.795 ± 0.221	7.347 ± 0.218

**Table 3:** Anterior eye relative axial distance changes with accommodation demand measured with the AS-OCT, compared to the Dubbelman model eye data [65]. The average age of the cohort has been matched to that of the model (19.4 years old; *n* = 30).

Accommodation Demand (D)	Dubbelman Model (mm)	AS-OCT (mm ± SD)
Anterior chamber depth (ACD)		
1	−0.040	−0.035 ± 0.038
2	−0.080	−0.079 ± 0.054
3	−0.120	−0.129 ± 0.055
4	−0.160	−0.173 ± 0.067
Lens Thickness (LT)		
1	0.048	0.037 ± 0.059
2	0.096	0.087 ± 0.070
3	0.144	0.170 ± 0.083
4	0.192	0.235 ± 0.091
Lens Centroid (ACD + LT/2)		
1	−0.016	−0.017 ± 0.029
2	−0.032	−0.035 ± 0.046
3	−0.048	−0.045 ± 0.045
4	−0.064	−0.056 ± 0.043
Anterior segment Length (ACD + LT)		
1	0.008	0.001 ± 0.044
2	0.016	0.008 ± 0.061
3	0.024	0.040 ± 0.067
4	0.032	0.062 ± 0.059
